# Contextual extension and validation of the Fo Mentality Scale among Chinese college students

**DOI:** 10.3389/fpsyg.2026.1850139

**Published:** 2026-08-18

**Authors:** Qing Chen, Zhongyi Xin

**Affiliations:** Faculty of Education, Shaanxi Xueqian Normal University, Xi'an, China

**Keywords:** Chinese sociocultural context, college students, Fo Mentality, psychometric validation, scale adaptation

## Abstract

Fo Mentality is a culturally embedded psychological orientation rooted in the Chinese sociocultural context and prevalent among contemporary young adults. Although labeled with the term “Fo” (Buddha), the construct represents a secular psychological orientation rather than Buddhist beliefs or religious practices. Existing measures of Fo Mentality have been developed primarily in workplace contexts, and whether this conceptualization applies to college students, whose experiences differ in academic demands, evaluation systems, and developmental tasks, remains unclear. The present study aimed to adapt and validate the Fo Mentality Scale for use among Chinese college students. Drawing on the conceptual framework and item content of the Workplace Fo Mentality Scale, qualitative analyses were conducted to assess contextual relevance and refine item wording for the university setting. A total of 1,166 college students were randomly divided into two independent subsamples for exploratory and confirmatory factor analyses, and an additional sample of 500 students was recruited to examine criterion-related validity. Results supported a stable four-factor structure comprising External Reward Detachment, Contentment with the Status Quo, Interpersonal Compliance, and Achievement Deprioritization. The adapted scale demonstrated satisfactory reliability and validity, and Fo Mentality showed positive associations with subjective well-being but negative associations with creativity. The findings extend the application of the Fo Mentality construct beyond workplace contexts and provide a psychometrically sound measure for assessing Fo Mentality among Chinese college students.

## 1 Introduction

With the accelerating process of social modernization and the rapid development of online media communication, Fo Mentality has gradually emerged and become widely disseminated among young people in recent years (Tian and Zhang, 2024; Wu, 2022). This phenomenon not only reflects the evolving social mentality of contemporary Chinese youth (Huang et al., 2024), but also influences psychological functioning and behavioral performance across multiple domains, including academic development, daily life, interpersonal interactions, and personal growth (Liu, 2023).

Although research on Fo Mentality has attracted increasing scholarly attention, existing studies remain largely at the level of theoretical interpretation and phenomenological description. Empirical investigations are still limited, with workplace Fo Mentality representing the only systematically operationalized research line to date (Yan et al., 2024).

Within this line of research, workplace Fo Mentality has been conceptualized as a context-dependent and malleable psychological orientation embedded within specific social environments and characterized by four dimensions: indifference to external rewards, contentment with the Status Quo, interpersonal harmony, and acceptance of the natural course of events. Building on this conceptualization, a psychometrically validated measurement scale has been developed, providing an important empirical foundation for operationalizing the construct (Yan et al., 2024).

However, despite these advances in conceptualization and measurement, the theoretical boundaries of Fo Mentality remain insufficiently specified, particularly regarding its conceptual position within the broader psychological literature. Clarifying its distinction from related motivational, coping, and contemplative psychological constructs is therefore essential for establishing Fo Mentality as a theoretically distinct construct.

Importantly, Fo Mentality should not be understood as a simple extension or repackaging of traditional motivational concepts or coping strategies. Rather, it can be conceptualized as a culturally embedded psychological orientation that shapes how individuals evaluate achievement, social comparison, and life experiences within a particular sociocultural context (Park, 2010). As a value orientation, Fo Mentality emphasizes psychological balance, inner harmony, and contentment, suggesting that individuals may derive meaning not only from continual achievement striving but also from maintaining internal equilibrium and satisfaction with their circumstances (Yan et al., 2024).

This conceptualization distinguishes Fo Mentality from motivation and coping, which represent more specific regulatory processes. Motivational constructs primarily concern the initiation, direction, and persistence of goal-directed behavior toward valued outcomes (Deci and Ryan, 2000; Locke and Latham, 2002), whereas coping strategies refer to cognitive and behavioral efforts aimed at managing demands appraised as stressful (Lazarus and Folkman, 1984). Thus, motivation and coping primarily operate as context-specific regulatory mechanisms, whereas Fo Mentality reflects a broader value orientation that influences how individuals evaluate and respond to achievement-related and social contexts.

Although Fo Mentality shares some similarities with constructs such as mindfulness and non-attachment, these constructs differ in their primary psychological functions. Mindfulness refers to an attentional process involving present-moment awareness and nonjudgmental acceptance of experiences (Kabat-Zinn, 2003), whereas non-attachment refers to a flexible relationship with experiences, desires, and outcomes characterized by reduced fixation on particular thoughts, goals, or self-related beliefs (Sahdra et al., 2010). In contrast, Fo Mentality focuses on how individuals evaluate achievement, competition, social comparison, and life goals within a culturally specific context. Therefore, mindfulness and non-attachment primarily describe processes involved in regulating internal experiences, whereas Fo Mentality concerns individuals’ value-based evaluations of social expectations and personal striving.

Given that Fo Mentality represents a culturally grounded value orientation, its specific manifestations may vary according to contextual demands and developmental tasks across different social environments. Accordingly, whether the workplace-based conceptualization and measurement framework adequately captures Fo Mentality among other populations, particularly college students facing distinct developmental challenges and social expectations, requires further empirical examination.

Building on this rationale, the present study aimed to adapt and validate a Fo Mentality Scale for Chinese college students.

## 2 Adaptation of the Fo Mentality Scale for college students

### 2.1 Theoretical conceptualization of the scale structure

The College Student Fo Mentality Scale was adapted from the Workplace Fo Mentality Scale. The original conceptual framework served as the basis for the present study and was subsequently evaluated and refined through qualitative and quantitative procedures within the college student population.

### 2.2 Open-ended questionnaire survey

To examine the applicability of the workplace-based conceptual framework and to identify expressions of Fo Mentality that were salient among college students, an open-ended questionnaire survey was conducted. A convenience sampling method was adopted, and 150 college students were recruited. After excluding invalid responses, 121 valid questionnaires were retained for analysis. The final sample included 50 males and 71 females, with a mean age of 20.16 ± 1.03 years.

The open-ended questionnaire included the following questions: “What do you think the ‘Fo’ Mentality is? What are its main characteristics?” and “How is your own ‘Fo’ Mentality manifested? What behaviors of yourself or your peers make you perceive them as having a Fo Mentality? Could you provide specific examples?”

### 2.3 Open coding

Two researchers with experience in qualitative research independently reviewed all 121 valid responses. A line-by-line coding procedure was adopted to extract key concepts and semantic units related to the Fo Mentality among college students. During coding, *in vivo* codes—participants’ own original expressions—were prioritized in order to minimize premature theoretical interpretation.

To ensure coding reliability, 30% of the responses were randomly selected for intercoder reliability testing. Both percentage agreement and Cohen’s Kappa were calculated. The results showed a percentage agreement of 87% and a Cohen’s Kappa of 0.73, indicating acceptable inter-coder reliability according to established qualitative research standards (Landis and Koch, 1977).

After the independent coding phase, the two coders and four additional researchers jointly discussed discrepancies and reached consensus on the initial coding scheme. This process resulted in 86 initial codes.

### 2.4 Selective coding

Following the initial coding process, six researchers collaboratively reviewed, categorized, and integrated all codes. Based on semantic similarities, logical relationships, and participants’ original narratives, the initial codes were grouped into 18 themes. These themes were then compared with the four-dimensional conceptual framework of workplace Fo Mentality (Yan et al., 2024). The findings generally supported the original four-dimensional structure; however, several dimension labels were revised to better capture the meanings emerging from the college student data. Consequently, four higher-order categories were retained while their conceptual labels were refined to enhance contextual relevance and content validity (see Table 1).

**Table 1:** Selective coding results (partial).

Representative raw quotations	Open codes	Core categories
People around me usually say I’m pretty easygoing. I do not really have much aggression, and they feel relaxed hanging out with me. No one has ever said I’m hard to get along with.	Easygoing	Interpersonal compliance
If someone asks me for help, I basically never say no. Over time, people just know I will not refuse. I guess I’ve kind of become the “nice guy” type.	Never say no	
When I’m with people, I’m not really afraid of conflict—it’s just that I do not think it’s necessary. Even if I feel a bit uncomfortable, I usually do not say anything. It’s not worth arguing over small stuff.	No need for conflict	
If I disagree with someone, I usually do not insist on my own opinion. Once the other person becomes more firm, I just give in.	Do not insist	
I do not really like arguing with people. Even if I think I’m right, I do not bother proving it.	No need to argue	
In group discussions, I usually do not speak up much. I do have ideas, but I just do not feel like saying them.	Do not speak up	Achievement deprioritization
I rarely go after opportunities on my own, even if I’m good at something. It feels kind of awkward to actively compete for things. And honestly, it does not even guarantee anything.	Do not go after	
Once I finish an assignment, I usually just leave it at that. As long as it passes, I do not really go back to improve it. It feels unnecessary.	Leave it at that	
I feel like I just do my best, and after that the result is out of my control. Whether I do well or not does not really bother me that much.	Does not really matter	
I do not usually sign up for competitions or activities unless a teacher assigns me. If it’s up to me, I probably would not bother.	Do not sign up	
Grades and rankings honestly do not matter that much to me. As long as I can graduate, that’s enough. I’ve never really cared about scholarships either.	Just graduate	External reward detachment
I do not really care how other people see me. Whether they think I’m unmotivated or “too Fo,” I do not bother explaining.	Do not care what others think	
I do not really think too much about gains and losses. Whether I get something or not does not affect me that much. I just go with the flow.	Go with flow	
Awards, points, and stuff like that do not really interest me. Other people might go after them, but I do not really care.	Do not care	
I’m actually pretty comfortable with how things are now. I do not really want to change anything because it feels like too much trouble to adjust.	Comfortable as is	Contentment with the Status Quo
I do not really have big ambitions. I never set goals like “I must be top of the class” or “I must get into a top company.” Just doing okay feels fine.	Just okay is fine	
When I go to a new environment, I do not feel much—no anxiety or excitement. I just adapt slowly and go with it.	Go with it	
I honestly do not have much career planning. I do not really know what I want to do in the future, and I’ll just figure it out later.	No planning	

Interpersonal Compliance refers to a tendency to maintain interpersonal harmony by avoiding unnecessary conflict and accommodating others during social interactions. Achievement Deprioritization reflects a relatively reduced emphasis on continuous achievement striving and upward mobility, indicating a less competitive orientation toward self-improvement and achievement-related goals. External Reward Detachment refers to an individual’s reduced emphasis on external recognition, material rewards, and achievement outcomes. Contentment with the Status Quo reflects a tendency to experience satisfaction with and acceptance of one’s current circumstances, with less emphasis on actively pursuing change or upward striving.

### 2.5 Item adaptation

Based on the qualitative coding results and the Workplace Fo Mentality Scale, the item pool was adapted and refined to reflect the linguistic expressions and experiences of college students. The initial adapted item pool comprised 18 items across four dimensions: External Reward Detachment (4 items), Contentment with the Status Quo (4 items), Interpersonal Compliance (5 items), and Achievement Deprioritization (5 items), which retained the original conceptual structure while incorporating context-specific modifications.

To ensure content validity, two doctoral-level psychologists were invited to evaluate the dimensional structure, item content, and wording of the scale. Their review focused on ensuring that each item adequately represented its intended theoretical dimension.

Prior to the formal survey, a pilot test was conducted with 50 college students. Based on participants’ feedback, several items were revised to improve clarity and accuracy. For example, some participants indicated that the original wording of Item 6, “With regard to opportunities, there is no need to force things regardless of whether one is good at them,” implied a fatalistic attitude. However, the intended meaning of the item was to capture a reduced emphasis on proactive striving and achievement-oriented self-improvement as central personal priorities, rather than fatalistic acceptance or a passive belief that outcomes are predetermined. Accordingly, the item was revised to: “Even when opportunities align with my strengths, I usually do not actively pursue them,” thereby improving the precision of the construct representation.

Following the pilot test and subsequent revisions, the final scale was developed. All items were rated on a 7-point Likert scale ranging from 1 (strongly disagree) to 7 (strongly agree). In addition, three validity-check items were included to identify careless responding and reduce the influence of social desirability bias. Responses to these items were used solely for data screening purposes and were not included in subsequent statistical analyses.

## 3 Validation of the Fo Mentality scale for college students

### 3.1 Participants

A convenience sampling method was used to recruit undergraduate students from six universities in Shaanxi Province, China. Data were collected via mobile-based questionnaires. A total of 1,300 questionnaires were distributed. After data screening procedures, including response time checks, detection of patterned responding, and removal of multivariate outliers, 134 cases were excluded. The final sample consisted of 1,166 valid responses, yielding an effective response rate of 89.69%. The valid sample was randomly split into two equal subsamples (50%/50%). Sample 1 (*n* = 583) was used for item analysis and exploratory factor analysis (EFA), while Sample 2 (*n* = 583) was used for confirmatory factor analysis (CFA) and construct validity testing.

Sample 1 included 271 males and 312 females, with a mean age of 20.41 ± 1.26 years. Sample 2 included 273 males and 310 females, with a mean age of 20.48 ± 1.51 years.

### 3.2 Data analysis

Data analyses were conducted using SPSS 27.0 and R (lavaan package, version 0.6–19). SPSS 27.0 was used for item analysis, descriptive statistics, correlation analysis, exploratory factor analysis (EFA), and regression analysis. Confirmatory factor analysis (CFA) was performed using the lavaan package in R to examine the structural validity of the scale.

#### 3.2.1 Item analysis

Item analysis was conducted using data from Sample 1 (*n* = 583). First, participants were ranked according to their total scale scores, and the upper and lower 27% groups were identified as the high-score and low-score groups, respectively. Independent-samples t-tests were conducted to examine differences between the two groups on each item. The results (see Table 2) indicated that the critical ratios (CRs) of the 18 items ranged from 5.01 to 15.36, and all differences between the two groups were statistically significant (*p* < 0.001).

**Table 2:** Corrected item–total correlations and independent-samples t-test results (sample 1, *n* = 583).

Item	*r*	*t*	Item	*r*	*t*	Item	*r*	*t*
1	0.24	5.01	7	0.50	10.990	13	0.39	12.497
2	0.54	13.19	8	0.50	11.559	14	0.47	13.391
3	0.42	10.73	9	0.59	14.92	15	0.43	9.81
4	0.47	11.37	10	0.52	12.78	16	0.46	9.98
5	0.50	11.28	11	0.39	9.55	17	0.46	10.15
6	0.62	15.36	12	0.27	6.19	18	0.57	14.27

All t-test results were significant at the *p* < 0.001 level.

Subsequently, corrected item–total correlations were calculated for each item. The results showed that item–total correlations ranged from 0.24 to 0.62. Items with correlations below 0.30 were considered to have weak consistency with the overall construct, indicating limited homogeneity with the scale. Retaining such items may reduce the internal consistency of the scale. Based on this criterion, items Q1 and Q12 were removed from subsequent analyses.

Finally, the internal consistency reliability of the scale was examined. The Cronbach’s *α* coefficient for the full scale in this sample was 0.80. After sequential deletion of each item, no improvement in α values was observed. These results suggest that 16 items met the minimum requirements for item retention.

#### 3.2.2 Exploratory factor analysis (EFA)

Exploratory factor analysis was conducted on the 16 retained items to examine the underlying dimensional structure of the scale. The suitability of the data for factor analysis was confirmed, with a Kaiser–Meyer–Olkin (KMO) value of 0.828 indicating good sampling adequacy and a significant Bartlett’s test of sphericity (χ^2^ = 2528.071, *p* < 0.001), supporting the appropriateness of factor extraction.

Principal Axis Factoring (PAF) with Promax rotation was employed to identify the latent factor structure of the scale. The initial analysis suggested a four-factor solution. However, four items (Q3, Q6, Q9, and Q18) exhibited substantial cross-loadings across multiple factors, indicating insufficient factorial clarity and weak discriminant validity. These items were therefore removed to improve the conceptual distinctiveness and psychometric quality of the scale.

A second PAF analysis was subsequently conducted on the remaining 12 items. The results supported a clear four-factor structure, accounting for 64.57% of the total variance. All items loaded consistently on their hypothesized dimensions (see Table 3), indicating a clear and interpretable factor structure.

**Table 3:** Factor loadings from principal axis factoring (PAF) of the college student Fo Mentality Scale (Sample 1, *n* = 583).

Item	Interpersonal compliance	Contentment with the Status Quo	External reward detachment	Achievement deprioritization
Q2 I rarely refuse others’ requests and am often seen as a “nice person.”	0.70			
Q4 When I disagree with others, I tend to give in.	0.70			
Q5 I rarely argue with others over right or wrong, or winning or losing.	0.69			
Q7 Even when opportunities match my strengths, I usually do not actively pursue them.				0.67
Q8 After achieving a goal, I do not want to put extra effort into further improvement.				0.68
Q10 I do not actively participate in competitions or strive for awards and recognition.				0.66
Q11 I do not care about rankings or scholarships.			0.69	
Q13 I do not overthink gains and losses in academic settings.			0.64	
Q14 I feel indifferent toward the university’s reward and incentive system.			0.68	
Q15 I feel comfortable with my current study status and do not want to change it.		0.71		
Q16 I have weak ambition and do not set very high goals for myself.		0.69		
Q17 I tend to go with the flow when facing new environments.		0.68		
Percentage of variance explained (%) 64.57%	24.23	14.40	13.06	12.88

### 3.3 Reliability analysis

Internal consistency reliability was examined using data from Sample 2 (*n* = 583). The Cronbach’s *α* coefficient for the overall scale was 0.77. The Cronbach’s α coefficients for the four dimensions were 0.75, 0.72, 0.75, and 0.77, respectively. These results indicate acceptable internal consistency reliability for the 12-item College Student Fo Mentality Scale, meeting psychometric standards.

### 3.4 Validity analysis

#### 3.4.1 Construct validity

Confirmatory factor analysis (CFA) was conducted using data from Sample 2 (*n* = 583) to examine the construct validity of the proposed four-factor model. Model fit was evaluated using multiple fit indices (see Table 4). The results indicated good model fit: χ^2^/df = 2.74, CFI = 0.96, TLI = 0.94, SRM*R* = 0.03, and RMSEA = 0.06. All indices met commonly accepted criteria, supporting the adequacy of the four-factor structure.

**Table 4:** Model fit indices (Sample 2, *n* = 583).

Model	χ^2^/df	CFI	TLI	SRMR	RMSEA
12-item model	2.74	0.96	0.94	0.03	0.06

Standardized factor loadings ranged from 0.63 to 0.79 (*p* < 0.001), indicating that all items significantly loaded on their respective latent factors and adequately represented the observed variables.

Convergent validity was further assessed using average variance extracted (AVE) and composite reliability (CR). The AVE values for Interpersonal Compliance, Contentment with the Status Quo, External Reward Detachment, and Achievement Deprioritization were 0.50, 0.53, 0.51, and 0.46, respectively. The corresponding CR values were 0.75, 0.77, 0.75, and 0.72. These results suggest that the revised College Student Fo Mentality Scale demonstrates acceptable convergent validity.

As shown in Table 5, the correlations among the four dimensions ranged from 0.212 to 0.363, all of which were lower than the square roots of the corresponding average variance extracted (AVE) values (0.705, 0.681, 0.711, and 0.728, respectively). According to the Fornell and Larcker (1981) criterion, this indicates that each construct shares more variance with its own indicators than with other constructs, supporting adequate discriminant validity. The results further suggest that while the dimensions are moderately correlated, they remain empirically distinct.

**Table 5:** Discriminant validity results.

Construct	Interpersonal compliance	Achievement deprioritization	External reward detachment	Contentment with the Status Quo
Interpersonal compliance	0.705			
Achievement deprioritization	0.352	0.681		
External reward detachment	0.307	0.348	0.711	
Contentment with the Status Quo	0.212	0.313	0.363	0.728

In addition, the correlations between each dimension and the total scale score were all above 0.60, indicating strong associations. This finding provides further evidence of construct coherence, suggesting that each dimension is consistent with the overall conceptual framework of the scale.

To examine measurement invariance across gender, multi-group confirmatory factor analysis (MG-CFA) was conducted with gender as the grouping variable (male = 273, female = 310).

The configural invariance model demonstrated good model fit (CFI = 0.962, RMSEA = 0.050, SRM*R* = 0.034), indicating that the four-factor structure of the scale was consistent across gender groups.

Subsequently, metric invariance was tested by constraining factor loadings to be equal across groups. The model fit showed negligible changes compared with the configural model (ΔCFI = 0.001, ΔRMSEA = 0.002), suggesting that participants across gender groups interpreted the items in a comparable manner, thus supporting metric invariance.

Next, scalar invariance was examined by additionally constraining item intercepts. The model fit remained stable with minimal changes (ΔCFI = 0.002, ΔRMSEA = 0.003), indicating that the scale demonstrated satisfactory parameter stability across groups, thereby supporting scalar invariance.

Taken together, according to commonly accepted criteria for measurement invariance (ΔCFI < 0.01 and ΔRMSEA < 0.015), the results provide evidence that the scale achieves measurement invariance across gender groups, supporting the validity of cross-group comparisons.

#### 3.4.2 Predictive validity

##### 3.4.2.1 Hypotheses development

Existing research suggests that Fo Mentality may have context-dependent implications. On the one hand, it reflects potentially adaptive orientations, including reduced emphasis on material pursuits, greater attention to internal experiences, self-reflection, and emotional calmness, which may contribute to psychological balance and social harmony (Cui et al., 2024; He, 2024; Li, 2019). On the other hand, when manifested as reduced emphasis on personal growth, achievement striving, or engagement with challenging goals, Fo Mentality may be associated with lower initiative and reduced involvement in achievement-related activities (Sun, 2021; Tan, 2021; Zheng and Fang, 2025). Therefore, the psychological consequences of Fo Mentality may depend on the specific contexts in which this orientation is activated and expressed.

From a theoretical perspective, the Multiple Discrepancies Theory posits that subjective well-being is influenced by perceived discrepancies between individuals’ actual circumstances and their internal standards, with smaller discrepancies generally associated with greater well-being (Michalos, 1985). Within this framework, Fo Mentality may contribute to subjective well-being by reducing the emphasis placed on external achievement standards and social comparison criteria, thereby decreasing perceived discrepancies between current circumstances and desired states. Consequently, individuals with a stronger Fo Mentality may experience greater satisfaction and psychological balance even when their objective circumstances remain unchanged (Zhang et al., 2025).

Moreover, the interpersonal compliance dimension of Fo Mentality may contribute to subjective well-being through its influence on interpersonal orientations. Specifically, this dimension reflects a tendency to prioritize interpersonal harmony, tolerance, and accommodation during social interactions. Prior studies suggest that individuals with stronger Fo tendencies are more likely to adopt low-conflict and high-tolerance interaction patterns in social contexts (Yan et al., 2024), which may reduce interpersonal strain and emotional burden and thereby contribute to greater psychological well-being.

In addition, the reduced emphasis on competitive achievement associated with Fo Mentality may help individuals manage psychological demands in high-pressure environments by reducing excessive concerns about comparison and performance expectations. Such an orientation may be associated with lower levels of stress-related experiences, including anxiety and burnout (Parker et al., 2022). Accordingly, the following hypothesis is proposed:

*H1:* Fo Mentality is positively associated with subjective well-being among college students.

However, the same orientation that promotes psychological balance and satisfaction may not necessarily facilitate outcomes requiring exploration, change, and persistent striving, such as creativity. Given that creativity plays an important role in adaptation and problem solving in dynamic environments (Baer and Oldham, 2006), identifying factors that may influence creative performance is particularly important among college students. Previous research has demonstrated that growth-related orientations and intrinsic motivational resources are important predictors of creativity (Shalley et al., 2009). However, Fo Mentality may reflect a value orientation that places less emphasis on continuous achievement striving and proactive change, while giving greater priority to acceptance of existing circumstances and psychological balance. Such an orientation may reduce individuals’ willingness to explore novel possibilities, engage with challenging tasks, and persist in uncertain situations, thereby constraining creative performance.

Furthermore, proactive personality has been widely identified as a robust predictor of creativity (Kickul and Gundry, 2002; Kim et al., 2009; Strauss et al., 2015), as proactive individuals are more likely to identify problems, seek novel information, and engage in experimentation, thereby fostering creative outcomes. In contrast, Fo Mentality may involve a reduced emphasis on proactive change and exploratory engagement across academic and daily contexts, reflecting a greater tendency to accept existing circumstances rather than actively pursue modification or advancement. Consistent with this perspective, empirical evidence from organizational settings has shown that Fo Mentality negatively predicts employee creativity (Yan et al., 2024). Although these findings were obtained among employees, they suggest that the value orientation underlying Fo Mentality may also influence creativity-related behaviors in other developmental contexts, such as college settings. Based on this theoretical and empirical evidence, the following hypothesis is proposed:

*H2:* Fo Mentality is negatively associated with creativity among college students.

##### 3.4.2.2 Procedure and sample selection

To minimize the potential impact of common method bias, data were collected anonymously using a two-wave survey design. At Time 1, undergraduate students from several universities in Shaanxi Province completed the Fo Mentality Scale via the Wenjuanxing online survey platform. One month later, at Time 2, the same participants were invited to complete measures of subjective well-being and creativity. After matching responses across the two waves, 500 valid questionnaires were retained for analysis. The final sample consisted of 214 males (42.8%) and 286 females (57.2%), with a mean age of 20.58 ± 0.93 years.

##### 3.4.2.3 Measures

###### Fo Mentality

Fo Mentality was assessed using the 12-item scale developed in the present study based on exploratory and confirmatory factor analyses reported above. Responses were rated on a 7-point Likert scale, with higher scores indicating a stronger level of Fo Mentality. In this study, the scale demonstrated acceptable internal consistency (Cronbach’s *α* = 0.76).

###### Subjective well-being

Subjective well-being was assessed using the General Subjective Well-Being subscale from the International College Survey (ICS) developed by Diener et al. (1999) and revised by Yang (2007). The scale consists of 5 items rated on a 7-point Likert scale, with higher scores indicating greater subjective well-being. The Cronbach’s α coefficient in the present study was 0.78.

###### Creativity

Creativity was assessed using the scale developed by Zhou and George (2001). The original English version was translated and back-translated following Brislin’s (1980) translation–back translation procedure to ensure cross-linguistic equivalence. The scale consists of 13 items rated on a 5-point Likert scale, with higher scores indicating higher levels of creativity. The Cronbach’s α coefficient in this study was 0.86.

##### 3.4.2.4 Results

Common method bias was addressed at the design, data collection, and statistical stages. At the design stage, different response formats were used and item order was randomized to reduce response patterns. At the data collection stage, a time-separated and batch-based anonymous survey procedure was adopted. At the statistical stage, Harman’s single-factor test was conducted to assess potential common method variance. The results indicated that six factors had eigenvalues greater than 1, and the first factor accounted for 16.3% of the total variance, which is below the recommended threshold of 40% (Podsakoff et al., 2003; Podsakoff et al., 2024). These results suggest that common method bias was not a serious concern in this study.

Correlation analyses showed that Fo Mentality was positively associated with subjective well-being (*r* = 0.36, *p* < 0.001) and negatively associated with creativity (*r* = −0.23, *p* < 0.001).

Regression analyses further indicated that Fo Mentality significantly and positively predicted subjective well-being (*β* = 0.36, *p* < 0.001), supporting Hypothesis 1, and significantly and negatively predicted creativity (*β* = −0.23, *p* < 0.001), supporting Hypothesis 2.

After controlling for gender, age, and academic discipline, the regression results remained stable and did not change substantially, indicating robust findings (see Table 6). In addition, the model explained 14% of the variance in subjective well-being and 6% of the variance in creativity. These results indicate moderate explanatory power for subjective well-being and modest explanatory power for creativity.

**Table 6:** Regression analysis results (*n* = 500).

Predictor	Subjective well-being				Creativity			
	*β*	SE	t	95%CI	*β*	SE	*t*	95%CI
Gender	−0.02	0.12	−0.44	[−0.29, 0.18]	0.03	0.11	0.72	[−0.13, 0.29]
Age	−0.01	0.07	−0.19	[−0.14, 0.12]	−0.01	0.06	−0.27	[−0.13, 0.10]
Academic discipline	−0.08	0.12	−1.81	[−0.45, 0.02]	−0.02	0.11	−0.54	[−0.26, 0.15]
Fo Mentality	0.37***	0.06	8.73***	[0.39, 0.61]	−0.23***	0.05	−5.28***	[−0.36, −0.17]
R^2^	0.14				0.06			
Adjusted R^2^	0.13				0.05			
F	19.86^***^				7.23.^***^			

注: ^***^p < 0.001.

## 4 Discussion

### 4.1 Theoretical implications

This study adapted the conceptual framework of Fo Mentality to the college student context and examined its dimensional structure through qualitative analysis and systematic scale adaptation procedures. The results largely supported the four-dimensional structure proposed in the Workplace Fo Mentality Scale (Yan et al., 2024), although several content-level refinements were identified within each dimension. These findings indicate that while the overall structure of Fo Mentality remains stable across contexts, its specific manifestations may vary according to the developmental tasks and lived experiences of college students.

Empirical evidence from the present study suggests that Fo Mentality has differential implications across psychological outcomes among college students, being positively associated with subjective well-being while negatively associated with creativity. These findings extend previous evidence from workplace settings (Yan et al., 2024) by demonstrating that the psychological consequences of Fo Mentality may vary across developmental contexts and domains of individual functioning. Together, these findings support the view that Fo Mentality represents a context-dependent psychological orientation rather than a uniformly adaptive or maladaptive characteristic.

Drawing on the appraisal perspective of coping theory (Lazarus and Folkman, 1984), one possible explanation for the positive association between Fo Mentality and subjective well-being is that this orientation may shape how individuals interpret and evaluate stressful or uncontrollable circumstances. By emphasizing acceptance of current situations and reducing excessive discrepancies between personal expectations and actual conditions, Fo Mentality may help individuals adjust their evaluations of life circumstances and reduce cognitive–emotional strain. Through this process, individuals may experience greater psychological equilibrium and satisfaction with their lives, which may contribute to enhanced subjective well-being (Huang et al., 2024; Qiu and Ding, 2024; Ruan and Yang, 2018; Song and Bie, 2022; Tong, 2023; Ye, 2022).

In contrast, creativity research suggests that creative performance relies heavily on sustained cognitive engagement, persistence, and deep problem processing (Amabile, 1988). From this perspective, Fo Mentality may involve a reduced emphasis on achievement-oriented goals and challenging pursuits when individuals encounter demanding tasks. Its four dimensions—External Reward Detachment, Contentment with the Status Quo, Interpersonal Compliance, and Achievement Deprioritization—reflect different aspects of this value orientation, which may collectively influence individuals’ engagement in cognitively demanding and exploratory activities. Consequently, individuals with stronger Fo tendencies may be less inclined to invest effort in generating novel ideas, exploring alternative solutions, and persisting through uncertain or challenging situations.

Beyond cognitive engagement, intrinsic motivation has been identified as a key antecedent of creativity (Amabile, 1996; Ghosh et al., 2020). Intrinsically motivated individuals are more likely to persist in challenging tasks, explore novel possibilities, and engage in experimentation. In contrast, Fo Mentality may involve a lower prioritization of achievement-related advancement and novelty seeking, which could reduce individuals’ tendency to invest effort in intrinsically rewarding but uncertain or demanding activities (Eisenberger and Aselage, 2008; Sun et al., 2023). Consequently, individuals with stronger Fo tendencies may show less engagement in exploration and experimentation, thereby constraining creative performance.

In addition to the direction of these associations, differences in explanatory power provide further insight into the functional role of Fo Mentality. Specifically, Fo Mentality accounted for a greater proportion of variance in subjective well-being than in creativity. This pattern is theoretically meaningful because subjective well-being is closely related to individuals’ cognitive appraisals of their life circumstances and their perceived ability to adapt to environmental conditions (Diener and Ryan, 2009). From this perspective, core features of Fo Mentality—such as acceptance of current circumstances, reduced reliance on external rewards, and lower prioritization of competitive achievement—may directly shape how individuals evaluate their lives and personal accomplishments. These evaluative tendencies may reduce psychological strain arising from unmet expectations and social comparison, thereby facilitating more favorable perceptions of one’s current situation and contributing to higher subjective well-being.

In contrast, creativity represents a complex outcome influenced by multiple cognitive, motivational, personality, and contextual factors (Amabile, 1996; Shalley et al., 2004). Although Fo Mentality may influence certain motivational orientations and behavioral tendencies relevant to creativity, its effects are likely to be more distal and less pronounced than those of more proximal creativity-related determinants.

Taken together, these findings provide broader theoretical implications for understanding the nature of Fo Mentality. Although the present study did not directly examine the underlying mechanisms linking Fo Mentality to subjective well-being and creativity, the findings suggest that Fo Mentality should not be interpreted as an inherently positive or negative orientation. Rather, its psychological implications may vary across outcomes depending on the appraisal processes, value priorities, and behavioral tendencies it shapes in different contexts. Future research should further investigate how Fo Mentality influences individuals’ evaluation of goals, responses to social expectations, and engagement with different types of activities across developmental and cultural settings.

### 4.2 Practical implications

The present findings have several practical implications for higher education and student psychological development. First, the positive association between Fo Mentality and subjective well-being suggests that this orientation may be linked to cognitive and emotional patterns that help students manage certain academic and social pressures. Accordingly, educators and university administrators should avoid interpreting Fo Mentality in purely negative terms and recognize its potential role in supporting students’ psychological adjustment, particularly in highly competitive environments characterized by intense performance demands.

Second, the negative association between Fo Mentality and creativity suggests that, although Fo Mentality may contribute to psychological adaptation, it may also be associated with reduced engagement in exploratory activities and creative pursuits. Universities may therefore benefit from creating learning environments that support students’ psychological well-being while simultaneously encouraging intellectual curiosity, active engagement, and creative exploration.

More specifically, fostering autonomy-supportive learning climates, providing opportunities for self-directed learning, promoting growth-oriented beliefs and behaviors, and establishing diverse pathways for achievement may help students maintain psychological well-being while reducing the potential constraints associated with limited engagement in change-oriented and exploratory activities. Such efforts may be particularly important for helping students balance adaptation to external pressures with the pursuit of long-term growth, innovation, and personal development.

### 4.3 Limitations and future directions

Although the adapted Fo Mentality Scale developed in this study demonstrates satisfactory reliability and validity, several limitations should be acknowledged.

Regarding the sample characteristics, although participants were recruited from multiple universities, all institutions were located within a single province of China. This geographic concentration may limit the generalizability of the findings to university students from other regions. Given potential regional differences in cultural values, educational environments, and socioeconomic conditions, further cross-regional validation is warranted. Future research should also examine measurement invariance across different regions and populations to strengthen evidence for the scale’s robustness and generalizability.

In terms of study design, the cross-sectional nature of the present study, while appropriate for scale adaption and validation, precludes causal inference and does not allow for examination of temporal stability. Consequently, test–retest reliability was not assessed. Future studies should adopt longitudinal designs to evaluate the stability of Fo Mentality over time and to more rigorously investigate its dynamic and causal relationships with related outcomes.

From a conceptual perspective, although the proposed four-factor structure captures important dimensions of Fo Mentality among college students, the psychological processes underlying its development and its associations with individual outcomes remain insufficiently understood. Future research should integrate additional theoretical perspectives to clarify how Fo Mentality emerges within sociocultural contexts and through which processes it influences psychological functioning across different domains.

## 5 Conclusion

The present study adapted and validated the Fo Mentality Scale for use among college students and demonstrated that the adapted instrument possesses satisfactory psychometric properties. The findings further indicate that Fo Mentality is positively associated with subjective well-being but negatively associated with creativity, supporting its differentiated associations across domains of psychological functioning. The adapted scale provides a valuable tool for future research on Fo Mentality in higher education and contributes to a better understanding of this culturally embedded psychological orientation among contemporary college students.

## Data Availability

The raw data supporting the conclusions of this article will be made available by the authors, without undue reservation.
